# Prevalence of myocardial scarring in congenital heart disease - comparison between left ventricular pressure and volume overload using a novel black-blood delayed enhancement imaging technique

**DOI:** 10.1186/1532-429X-18-S1-P187

**Published:** 2016-01-27

**Authors:** Sihong Huang, Michael J Campbell, Piers C Barker, Han W Kim, David C Wendell, Elizabeth Jenista, Michele Parker, Stephen Darty, Brenda D Hayes, Enn-Ling Chen, Raymond Kim

**Affiliations:** grid.26009.3d0000000419367961Medicine/Cardiology DCMRC, Duke University, Durham, NC USA

## Background

Congenital heart disease (CHD) can result in left ventricular pressure and/or volume overloaded states, which may result in myocardial scarring. Prior studies have suggested that the presence of myocardial scar may be associated with adverse outcome in patients with CHD. The current gold standard for scar imaging is delayed-enhancement MRI (DE-MRI). However, it may be difficult to distinguish hyperenhanced subendocardial scars from the bright blood-pool with DE-MRI. We have developed a new, **F**low-**I**ndependent **D**ark-blood **D**e**L**ayed **E**nhancement technique (FIDDLE) that increases the conspicuity of subendocardial hyperenhancement, by making the blood-pool black. In this study we investigated the prevalence of myocardial scarring as determined by DE-MRI and FIDDLE in CHD patients with LV pressure or volume overload.

## Methods

Patients with CHD and LV pressure (n = 10) or volume overload (n = 11) were prospectively enrolled. Pressure overload required the presence of LV wall hypertrophy along with evidence of significant downstream obstruction (e.g. moderate to severe aortic stenosis, coarctation). Volume overload required the presence of moderate or severe LV ventricular dilation along with evidence of significant aortic or mitral regurgitation. Patients with asymptomatic bicuspid AV with normal LV size and wall thickness without significant valvular stenosis or regurgitation were enrolled as controls (n = 4). Standard DE-MRI and FIDDLE were acquired in each patient 10-20 minutes after gadolinium administration (0.15 mmol/kg), using matched parameters (eg. slice thickness, 7 mm; inplane resolution, 1.5 × 1.4 mm; temporal resolution; etc). DE-MRI and FIDDLE images were analyzed independently using a 17-segment model and a 5-point scale for scar transmurality.

## Results

**Figure 1:**
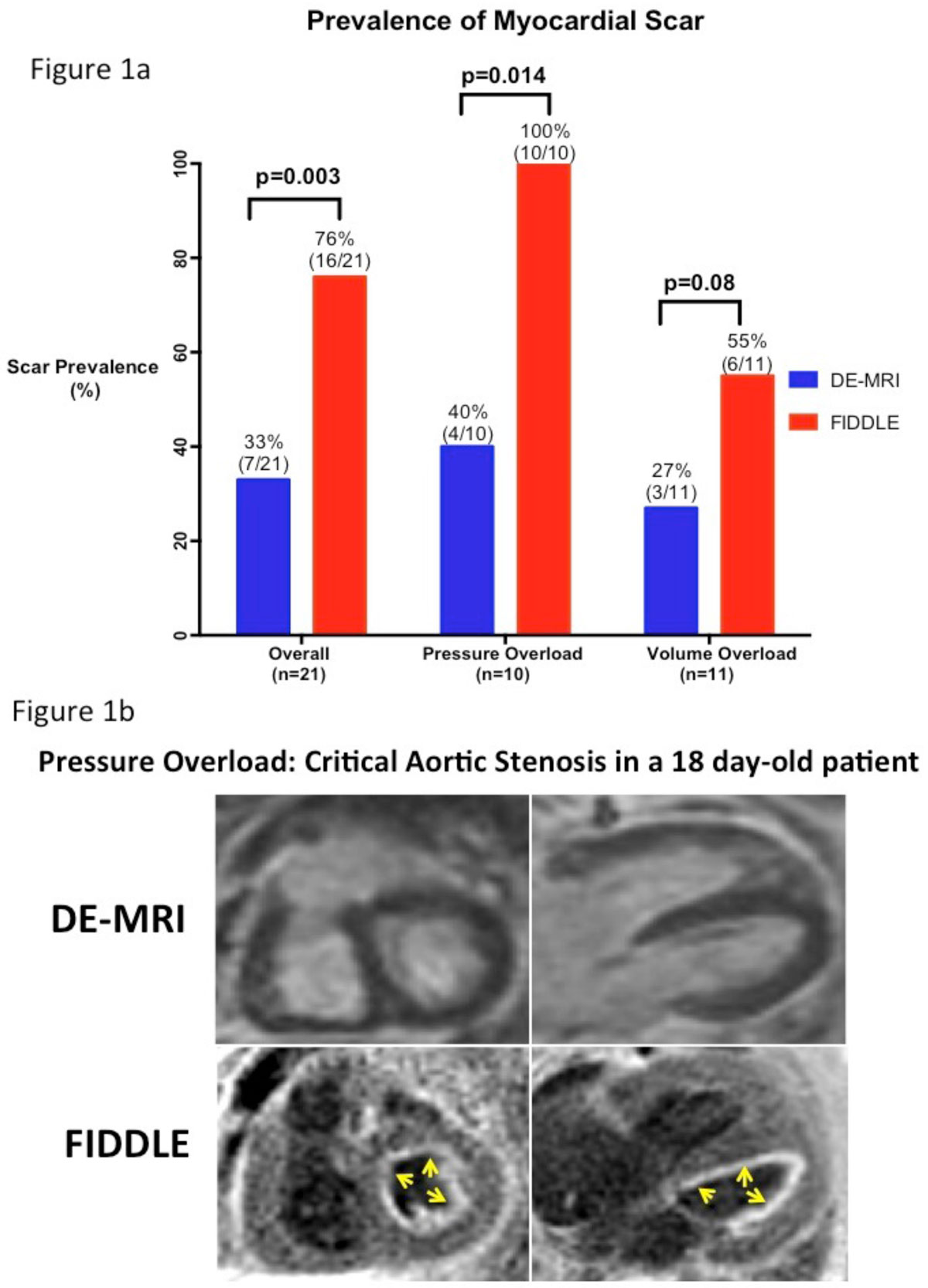
**Figure 1a: prevalence of myocardial scarring identified by FIDDLE (red) is significantly higher (p=0.003) than that by DE-MRI (blue)**. Figure 2b: global sub-endocardial hyperenhancement (arrows) identified by FIDDLE but not by DE-MRI in a 18 day-old patient with critical aortic stenosis.

Characteristics of the 3 cohorts are shown in the Table 1. In the 4 control patients, neither FIDDLE nor DE-MRI identified any myocardial scarring. In patients with pressure or volume overload, 16 (76%) and 7 (33%) patients were identified with scar by FIDDLE and DE-MRI, respectively (p=0.003; Figure 1a). The increase in prevalence of scar as identified by FIDDLE was primarily due to patients with pressure overload for which FIDDLE showed 100% (10/10) with subendocardial scarring. In patients with volume overload, the prevalence of scar was also found to be higher by FIDDLE than by DE-MRI, however, this did not reach statistical significance. An example of myocardial scarring seen by FIDDLE, but missed by DE-MRI is shown in **Figure 1b**. Note, the scar is nearly globally subendocardial throughout the LV (arrows). Even in patients with scar identified by DE-MRI, scar size was larger by FIDDLE compared with DE-MRI (p=0.0076).

**Table 1:** Characteristic of 3 cohorts

	Pressure Overload (n = 10)	Volume overload (n = 11)	Control (n = 4)
Diagnosis	Aortic stenosis (7) Coarctation (2) Shone's Complex (1)	Aortic Stenosis s/p Ross (3) Double inlet LV s/p Fontan (3) Truncus arteriosus s/p repair (1) D-TGA s/p arterial switch (1) ALCAPA with mod MR (1) Bicuspid AV with mod AI (1) Ebstein's malformation (1)	Bicuspid AV, asymtomatic
Mean LVEF	65 +/- 8%	52 +/-13%	67 +/- 6%
Mean scar size (by FIDDLE)	6.3 +/- 4%	6.7 +/-13%	0%

## Conclusions

Myocardial scarring is common in congenital heart disease patients with LV volume overload and ubiquitous in patients with LV pressure overload. FIDDLE is significantly more sensitive than DE-MRI at detecting subendocardial scarring.

